# Sustainable Thermoplastic Material Selection for Hybrid Vehicle Battery Packs in the Automotive Industry: A Comparative Multi-Criteria Decision-Making Approach

**DOI:** 10.3390/polym16192768

**Published:** 2024-09-30

**Authors:** Mustafa Sefa Bulut, Muhammed Ordu, Oguzhan Der, Gokhan Basar

**Affiliations:** 1 Department of Mechanical Engineering, Faculty of Engineering and Natural Sciences, Osmaniye Korkut Ata University, 80010 Osmaniye, Türkiye; mustafasefabulut@gmail.com; 2 Department of Industrial Engineering, Faculty of Engineering and Natural Sciences, Osmaniye Korkut Ata University, 80010 Osmaniye, Türkiye; gokhanbasar@osmaniye.edu.tr; 3 Department of Marine Vehicles Management Engineering, Faculty of Maritime, Bandirma Onyedi Eylul University, 10200 Balıkesir, Türkiye; oder@bandirma.edu.tr

**Keywords:** material selection, multi-criteria decision-making, automotive industry, hybrid vehicle battery packs, thermoplastics, polymer

## Abstract

This research study employs a comparative Multi-Criteria Decision-Making (MCDM) approach to select optimal thermoplastic materials for hybrid vehicle battery packs in the automotive industry, addressing the challenges posed by high-temperature environments. Through a detailed evaluation of materials based on criteria such as thermal stability, mechanical strength, chemical resistance, and environmental impact, the research identifies materials that enhance battery efficiency, longevity, and vehicle performance. Utilizing SWARA-ARAS, SWARA-EDAS, and SWARA-TOPSIS methods, the study systematically assesses and ranks various polymers, providing recommendations that prioritize safety, performance, and sustainability. The findings offer valuable insights for manufacturers in making informed material selection decisions, contributing to the advancement of sustainable automotive technologies. This research not only highlights the importance of material selection in the context of hybrid vehicle battery packs but also sets a foundation for future studies to explore emerging materials and decision-making frameworks, aiming to further enhance the efficiency and sustainability of hybrid vehicles.

## 1. Introduction

High-temperature applications, essential in the aerospace, automotive, energy, and manufacturing sectors, involve operations at temperatures beyond standard conditions, necessitating materials with high thermal resistance and stability [1,2,3]. These materials must endure extreme thermal stress, maintaining performance during thermal cycling [3,4]. Key applications include jet engine components, heat exchangers, furnaces, and boilers, with system performance critical to withstand thermal degradation and oxidation [1,2,4]. In high-temperature applications, selecting materials that endure extreme heat, corrosion, and thermal stress is essential, as evidenced in aerospace turbine blades and automotive exhaust systems [5,6,7]. Effective thermal insulation and cooling systems are integral for managing heat and ensuring component longevity and safety [8].

In the automotive industry, high-temperature concerns in hybrid vehicle battery packs, mainly lithium-ion, arise from the heat produced during operation and charging, risking thermal runaway and instability [9,10]. Effective thermal management through advanced cooling systems is essential to maintain battery performance, safety, and longevity [11]. Selecting polymer materials that withstand high temperatures and aid thermal regulation is crucial. Material choice for hybrid vehicle battery packs is critical, impacting safety, performance, and durability. These materials must resist high temperatures, thermal expansion, and mechanical stress from charging cycles, while also being sustainable [12,13]. Optimizing factors like thermal properties, strength, weight, and environmental impact is key to enhancing battery life and safety, underlining the complexity of material selection in hybrid vehicle manufacturing [14].

Multi-Criteria Decision-Making (MCDM) is crucial in decision-making, especially in selecting polymer materials for hybrid vehicle battery packs, balancing factors like thermal resistance, mechanical strength, and sustainability [15,16]. This research applies MCDM to identify optimal materials, integrate economic and environmental considerations, and enhance hybrid vehicle technology [17].

The objective of this study is to employ a comprehensive Multi-Criteria Decision-Making (MCDM) approach to select the most suitable polymer materials for hybrid vehicle battery packs in the automotive industry. This research addresses critical challenges such as thermal management, mechanical strength, chemical resistance, and environmental impact, which are vital for enhancing the efficiency, safety, and sustainability of hybrid vehicles. Specifically, the study applies the SWARA-ARAS, SWARA-EDAS, and SWARA-TOPSIS methods to systematically assess and rank various thermoplastic materials. The aim of the paper is to provide a detailed evaluation framework that not only aids in the selection of materials that can withstand high-temperature environments but also contributes to the overall performance and longevity of hybrid vehicle battery packs. By integrating economic and environmental considerations, this research offers valuable insights for manufacturers to make informed decisions in material selection, thus advancing sustainable automotive technologies. Furthermore, this study sets a foundation for future research to explore emerging materials and decision-making frameworks, aiming to further optimize the material selection process in the context of hybrid vehicles. Therefore, the study analyzes 10 polymer materials against 10 criteria, employing the SWARA method for weight assignment and ARAS, EDAS, and TOPSIS for comparative analysis. The main objective of comparing the three different MCDM methods (i.e., ARAS, EDAS, and TOPSIS) is to validate the rankings of alternatives derived from these decision-making processes and to show the consistency among these methods. Therefore, the consistency of these methods is verified through Spearman’s rank correlation, offering a systematic approach to material selection and advancing automotive material science. The findings provide insights into material suitability for battery packs, concluding with the study’s contributions and future research directions, ensuring a systematic exploration of material selection in hybrid vehicle technology.

## 2. Literature Review

MCDM methods have been successfully implemented in various sectors, demonstrating their ability to handle complex decision-making. For instance, in green supply chain management and green logistics, studies by Güner and Cebeci [18] and Agarwal et al. [19] underline the integration of sustainability in business operations. Research in healthcare by Ordu et al. [20] and renewable energy by Moosivand et al. [21] and García-Orozco et al. [22] showcases MCDM’s role in addressing critical issues like drug shortages and sustainable energy development. Additionally, studies by Raut et al. [23], Göncü and Çetin [24], and Van Nguyen et al. [25] illustrate MCDM’s application in areas like supplier selection in healthcare, blockchain in supply chain management, and sustainable logistics, highlighting its utility in varied complex scenarios [26].

In the realm of material selection, MCDM’s effectiveness is well-documented across industries and the application of MCDM techniques in material selection is essential for managing the complexity of decision-making processes that involve multiple criteria. These methods (i.e., ARAS, EDAS, and TOPSIS) provide significant flexibility and accuracy in determining the weights of decision criteria and ranking material alternatives [27,28]. The integrated use of these methods enhances the accuracy of the material selection process and supports the development of sustainable, high-performance solutions for demanding applications [29]. Emovon and Oghenenyerovwho [30] reviewed extensive literature, revealing a preference for hybrid MCDM techniques in material selection. Key contributions by Chatterjee and Chakraborty [31] include developing hybrid methods for sustainable material selection and its application in mechanical components, respectively. Moreover, Mathiyazhagan et al. [32] emphasized sustainability in construction material selection, showcasing MCDM’s role in achieving sustainable outcomes in diverse sectors.

The selection of sustainable materials for high-temperature environments is critical, especially in applications such as hybrid vehicle battery packs. These environments require polymer materials with exceptional durability, chemical resistance, and thermal conductivity. A sustainable approach must consider not only the performance characteristics but also the environmental impacts throughout the material’s lifecycle. Multi-Criteria Decision-Making (MCDM) methods, such as SWARA, EDAS, and TOPSIS, have been effectively employed to identify the most suitable materials for such challenging conditions [33,34,35]. These methods collectively allow for a comprehensive decision-making process that optimizes both technical performance and environmental sustainability [36,37].

This study enhances the literature by applying MCDM to thermoplastic material selection for hybrid vehicle battery packs, addressing operational challenges at high temperatures. It provides a novel perspective on comparative material analysis, highlighting the balance of criteria like thermal stability, cost, and environmental impact. This research offers a detailed framework for material selection in hybrid vehicles, contributing to material science and supporting sustainable advancements in automotive technology. While the selection of appropriate thermoplastic materials for hybrid vehicle battery packs has gained significant attention in recent research, most studies tend to focus on a limited set of criteria or employ a single decision-making method. This approach does not fully capture the multidimensional nature of the material selection process, leaving critical gaps in the decision-making framework. Specifically, there is a lack of comprehensive comparison that considers the environmental impacts, sustainability factors, and long-term performance of thermoplastic materials. This study aims to address this knowledge gap by employing a Multi-Criteria Decision-Making approach to systematically evaluate and rank thermoplastic materials, thereby providing more holistic and informed recommendations for material selection in hybrid vehicle battery packs.

## 3. Material and Methods

### 3.1. Material Alternatives

**Figure 1:**
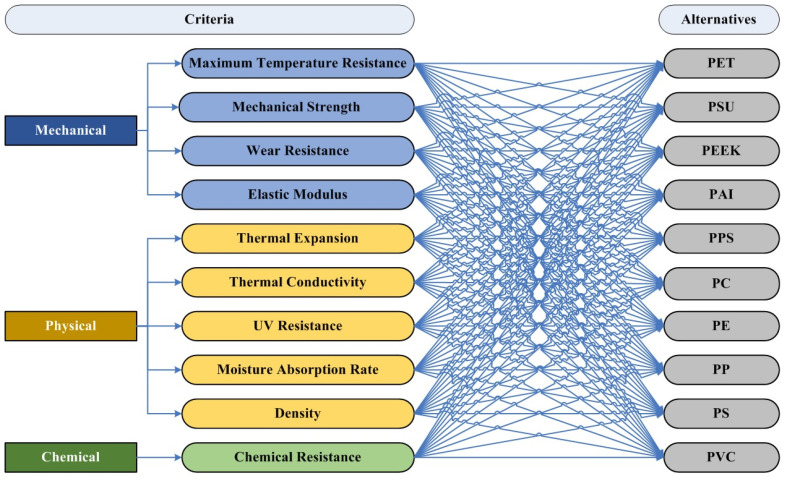
The hierarchical structure of the optimization problem.

In the selection of polymer materials for hybrid vehicle battery packs within the automotive industry, an in-depth evaluation of polymer materials is essential. This evaluation focuses on their thermal stability, mechanical properties, and their ability to meet the rigorous demands of hybrid vehicle operations (see Figure 1). Polyethylene Terephthalate (PET) is well-known for its outstanding mechanical and thermal stability characteristics. It provides a robust solution for components requiring resilience to thermal degradation and dimensional stability, thereby promising to enhance the structural integrity and longevity of battery packs [38]. Polysulfone (PSU) is characterized by its exceptional high-temperature resistance and mechanical strength. It serves as an ideal choice for parts exposed to elevated temperatures, leading to improved thermal management and extended durability of the system [39]. Polyetheretherketone (PEEK) stands out for its outstanding thermal resistance and mechanical strength. Suitable for demanding applications, it significantly elevates performance standards by offering superior resistance to thermal and mechanical stresses [40]. Polyamide-imide (PAI) exhibits remarkable high-temperature performance and dimensional stability. This is crucial for critical components requiring consistent reliability under thermal stress, ensuring the safety and operational efficiency of battery packs [41]. Polyphenylene Sulfide (PPS) is notable for its excellent chemical resistance and thermal stability. It is particularly suited for environments demanding resistance to corrosive substances and high temperatures, enhancing the battery pack’s durability and performance [42]. Polycarbonate (PC) offers high impact strength and thermal resistance. This makes it an attractive option for protecting components against physical impact, thereby improving safety features and resistance to thermal stresses within the battery pack [43]. Polyethylene (PE) is valued for its superior electrical insulation properties and chemical resistance. Ideal for insulating components, it enhances the electrical safety and performance of the system [44]. Polypropylene (PP) presents a good balance of chemical resistance, thermal stability, and mechanical properties [45]. It addresses the operational demands of hybrid vehicle battery packs, contributing to the system’s longevity and reliability. Polystyrene (PS) is suitable for non-critical parts of the battery pack where high temperatures are not a major concern. It offers cost-effective solutions for certain components with its ease of processing and dimensional stability [46]. Lastly, Polyvinyl Chloride (PVC) is known for its excellent electrical insulation properties and chemical resistance. Suitable for electrical insulation and protective sheathing, it enhances the overall safety and durability of the battery pack [47]. Through a Multi-Criteria Decision-Making process, this selection aims to identify materials that not only withstand high-temperature environments but also contribute to the performance, safety, and sustainability of hybrid vehicle battery packs.

### 3.2. Criteria

The selection process for polymer materials in hybrid vehicle battery packs within the automotive industry requires a holistic evaluation of several critical criteria (see Figure 1), each playing a key role in determining the suitability of materials under the rigorous operational demands of such applications. This evaluation ensures that the chosen polymer materials can withstand the challenges posed by hybrid vehicle environments, optimizing performance, safety, and durability. Firstly, the maximum temperature resistance and Coefficient of Thermal Expansion (CTE) are paramount. High-temperature resistance is crucial for the longevity and reliability of materials used in hybrid vehicle battery packs. Materials that can withstand extreme temperatures reduce the frequency of replacements and maintenance, thereby lowering resource consumption and waste generation. Moreover, by preventing thermal runaway—a phenomenon that can lead to catastrophic failures—these materials contribute to the safety and sustainability of the technology, reducing environmental risks associated with battery fires [48]. Low thermal expansion is essential to ensure that materials do not deform or fail under fluctuating temperatures. Materials with low coefficients of thermal expansion maintain their structural integrity across a wide temperature range, reducing the risk of mechanical failures. This stability is critical for the longevity and reliability of battery packs, which supports sustainable vehicle operation by reducing maintenance needs and associated environmental impacts [49]. Secondly, density and mechanical strength considerations are essential for optimizing the vehicle’s performance and efficiency. The density of a material affects the overall weight of the vehicle, which in turn impacts fuel efficiency and energy consumption. Lightweight materials contribute to reducing the vehicle’s energy requirements, thereby lowering greenhouse gas emissions. By selecting materials with an optimal balance of density and strength, the sustainability of the vehicle is enhanced through improved fuel economy and reduced environmental impact [50]. Materials with high mechanical strength ensure durability and structural integrity under various stress conditions, such as vibrations and impacts. This durability reduces the need for frequent part replacements, conserving resources and energy that would otherwise be used in the production and transportation of replacement parts. Additionally, strong materials enhance the overall safety of the vehicle, contributing to the sustainability of the automotive industry by extending the service life of components [51]. Furthermore, the elastic modulus and wear resistance of materials are important for their ability to absorb impacts and resist mechanical wear. A material’s elastic modulus indicates its ability to maintain shape and functionality under stress. Materials with an appropriate balance of stiffness and flexibility ensure that components can endure operational stresses without permanent deformation. This characteristic is vital for maintaining the efficiency and reliability of hybrid vehicle systems, reducing the need for repairs and replacements, and thus supporting sustainability [51]. Wear-resistant materials extend the operational life of components by minimizing the degradation that occurs during regular use. This resistance to wear reduces the frequency of replacements, which in turn conserves raw materials and energy. By enhancing the durability of components, wear resistance directly supports sustainable manufacturing practices and contributes to the reduction in waste [52].

Chemical resistance, moisture absorption rate, and thermal conductivity. The ability of a material to resist chemical degradation, particularly from corrosive substances like battery electrolytes, is critical for sustainable operation. Chemically resistant materials prolong the life of battery packs and other components by minimizing corrosion-related failures. This not only reduces hazardous waste but also decreases the environmental footprint of producing and disposing of replacement parts [53]. Low moisture absorption is essential for maintaining the material’s properties in varying environmental conditions. Materials with low moisture absorption resist swelling, corrosion, and other moisture-related damage, which prolongs their service life and reduces the frequency of replacements. This contributes to sustainability by minimizing waste and the environmental impact associated with frequent material replacements [54]. Proper thermal conductivity is crucial for effective heat management within battery packs. Polymer materials that either dissipate or insulate heat efficiently help maintain optimal operating temperatures, preventing overheating and extending the life of the battery. By optimizing thermal management, these materials contribute to the overall sustainability of hybrid vehicles by enhancing energy efficiency and reducing the likelihood of thermal-induced failures [55]. Lastly, Materials exposed to sunlight must resist UV-induced degradation to ensure long-term durability. UV-resistant materials maintain their structural integrity and performance over time, reducing the need for replacements and the associated environmental impact of producing new parts. This resistance is particularly important for external components that are frequently exposed to sunlight, supporting the sustainability of hybrid vehicles through improved material longevity [56].

For the ranking and selection outcomes of decision-making processes to be strong and dependable, it is vital to select appropriate criteria and apply both subjective and logical weighting to them. In this context, the study utilized the SWARA method—a subjective approach to weighting criteria. The criteria were assigned weights through a collaborative assessment by a group of experts, including mechanical and industrial engineers as well as academics in the field. Each of these criteria has been carefully selected to ensure that the materials chosen not only meet the technical requirements of hybrid vehicle battery packs but also contribute to broader sustainability goals. By optimizing these factors, we aim to support the development of hybrid vehicles that are not only efficient and safe but also environmentally responsible.

In summary, the MCDM process for selecting polymer materials for hybrid vehicle battery packs involves a balanced evaluation of these criteria, considering their impact on the performance, safety, and sustainability of the battery system. This comprehensive approach ensures that the selected polymer materials meet the high standards required for the efficient and safe operation of hybrid vehicles. The units and abbreviations of the criteria are given in Table 1.

### 3.3. A Comparative Multi-Criteria Decision-Making Approach

**Figure 2:**
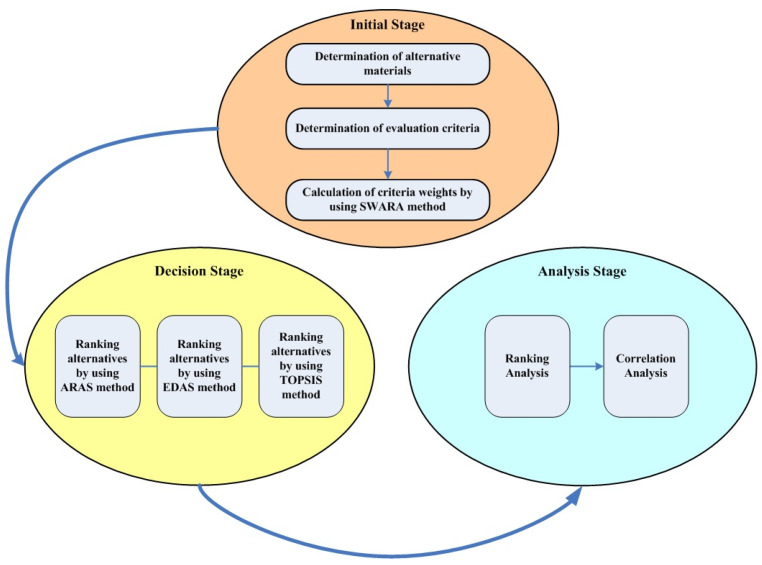
The structure of the approach used in the study.

A three-stage approach (see Figure 2) was adopted in the thermoplastic material selection process for hybrid vehicle battery packs. The first stage, also known as the initial stage, includes the determination of alternative thermoplastics suitable for the manufacturing of hybrid vehicle battery packs in terms of their chemical, mechanical, and physical properties, the determination of the criteria suitable for this selection process, and, finally, the calculation of the criterion weights, taking into account the relative advantages of the criteria over each other. The second stage, the decision stage, involves the ranking of alternative materials using three distinct MCDM methods: ARAS, EDAS, and TOPSIS. Later, the criteria were weighted by employing the SWARA approach in the first stage, they were embedded in MCDM methods in the decision stage. The last is the analysis stage, which consists of ranking analysis and correlation analysis, which reveals to what extent the rankings produced by these three distinct MCDM approaches are compatible with each other.

In the decision stage of the study, three distinct MCDM methods, namely ARAS, EDAS, and TOPSIS, were chosen to evaluate and rank the thermoplastic materials for hybrid vehicle battery packs. The selection of these methods is based on their proven effectiveness in handling complex decision-making problems, particularly in scenarios involving multiple and often conflicting criteria. The ARAS (Additive Ratio Assessment) method is favored for its capability to incorporate the utility function of alternatives directly, allowing for a straightforward and comprehensive comparison [57]. The EDAS (Evaluation based on Distance from Average Solution) method is chosen for its robustness in handling both benefit and cost criteria, which aligns well with the diverse set of criteria considered in this study [58]. TOPSIS (Technique for Order Preference by Similarity to Ideal Solution) is selected due to its intuitive approach of ranking alternatives based on their proximity to an ideal solution, which facilitates the decision-making process [59]. These MCDM methods have been successfully applied to various decision-making problems across different fields. For instance, the ARAS has been utilized in environmental management and supplier selection, demonstrating its versatility in different contexts. The EDAS has proven effective in inventory classification and sustainable energy planning, making it a robust choice for evaluating materials based on multiple criteria. The TOPSIS, widely recognized for its simplicity and effectiveness, has a long history of application in material selection and project management. Given their successful application in such complex decision environments, these methods were selected to ensure a comprehensive and reliable evaluation of thermoplastic materials in our study.

#### 3.3.1. Step-Wise Weight Assessment Ratio Analysis (SWARA) Approach for Weighting Criteria

The SWARA method is employed as an MCDM technique for establishing criterion weights. The steps of this method, as outlined by Keršulienė et al. [60], are provided below:

*Step 1:* The significance of the criteria is prioritized in a descending manner.

*Step 2:* To determine the Comparative Significance of the Mean Value (*s_j_*) for each criterion, criterion *j* is compared with criterion (*j* + 1). The relative significance of criterion *j* in comparison to criterion (*j* + 1) is then established.

*Step 3:* The coefficient (*k_j_*) is computed by using Equation (1).

(1) $$k_{j}=\left\{\begin{array}{cc} 1, & j=1\\ s_{j}+1, & j>1 \end{array}\right.$$

*Step 4:* Equation (2) is employed to calculate the importance vector (*q_j_*).

(2) $$q_{j}=\left\{\begin{array}{cc} 1, & j=1\\ \frac{k_{j-1}}{k_{j}}, & j>1 \end{array}\right.$$

*Step 5:* The criterion weights (*w_j_*) are determined by using Equation (3).

(3) $$w_{j}=\frac{q_{j}}{\sum_{k=1}^{n}q_{k}}$$

#### 3.3.2. Additive Ratio Assessment (ARAS) Approach

The standard MCDM issue involves the process of prioritizing a set of decision options, each defined by various decision factors that must be considered concurrently. According to the ARAS approach, the utility function value, which assesses the overall relative effectiveness of a feasible option, is directly connected to the comparative influence of the values and weights assigned to the main criteria evaluated within a project. The ARAS method consists of the following steps [57]:

*Step 1:* An initial decision matrix is developed with alternatives in the row and criteria in the column. The first row contains the optimum alternative values for the criteria. These values, whichever alternative meets the most ideal value of the relevant criterion, are considered as the most optimum value for that criterion. The optimal value of the criterion *j* (*x*_0*j*_) is calculated according to Equation (4).

(4) $$x_{0j}=\left\{\begin{array}{c} \underset{i}{\max}x_{ij},\;\;\;\;\;\;\;\mathrm{If}\;\mathrm{the}\;\mathrm{criterion}\;j\;\mathrm{is}\;\mathrm{maximization}-\mathrm{oriented}\\ \underset{i}{\min}x_{ij},\;\;\;\;\;\;\mathrm{If}\;\mathrm{the}\;\mathrm{criterion}\;j\;\mathrm{is}\;\mathrm{minimization}-\mathrm{oriented} \end{array}\right.$$

*Step 2:* After developing the initial decision matrix, the normalization procedure is carried out. Accordingly, Equation (5) is used for the minimization-oriented criteria or Equation (6) is applied for the maximization-oriented criteria.

(5) $${\bar{x}}_{ij}=\frac{x_{ij}}{\sum_{i=0}^{m}x_{ij}}$$

(6) $${\bar{x}}_{ij}=\frac{1/x_{ij}}{\sum_{i=0}^{m}{1/x}_{ij}}$$

where *x_ij_* means the value of the alternative *i* of the criterion *j* and ${\bar{x}}_{ij}$ is the normalized *x_ij_*.

*Step 3:* Subsequently, following the normalization procedure, the decision matrix is weighted using Equation (7).

(7) $${\hat{x}}_{ij}={\bar{x}}_{ij}w_{j}$$

where ${\hat{x}}_{ij}$ represents the value of the weighted ${\bar{x}}_{ij}$, and *w_j_* is the weight of the criterion *j*.

*Step 4:* For each alternative, the optimality function (*S_i_*) value and the degree of alternative utility (*K_i_*) are calculated by using Equations (8) and (9). *S*_0_ is the optimality function of the ideal alternative based upon which all criteria can have the most optimal value,

(8) $$S_{i}=\sum_{j=1}^{n}{\hat{x}}_{ij}$$

(9) $$K_{i}=\frac{S_{i}}{S_{0}}$$

#### 3.3.3. Evaluation Based on Distance from Average Solution (EDAS) Approach

The technique known as the EDAS employs the average solution as a basis for evaluating alternatives. It incorporates two metrics termed PDA (Positive Distance from Average) and NDA (Negative Distance from Average). These metrics are determined based on the nature of the criteria being either beneficial or non-beneficial. When dealing with conflicting criteria, this method proves highly beneficial. The preferred alternative exhibits a shorter distance from the ideal solution and a greater distance from the nadir solution. The steps of the EDAS approach are explained as follows [58]:

*Step 1*: The initial decision matrix is established.

*Step 2*: The average value (*AV*) is computed for each criterion using Equation (10). *n* is the number of the criteria:

(10) $${AV}_{j}=\frac{\sum_{i=1}^{n}X_{ij}}{n}$$

*Step 3*: The positive (*PDA*) and negative distance (*NDA*) values from the average are calculated based on the classification of criteria into benefit and cost type. Equations (11) and (12) are used for maximization-oriented criteria whereas Equations (13) and (14) are applied for minimization-oriented criteria.

(11) $${PDA}_{ij}=\frac{max(0,(X_{ij}-{AV}_{j}))}{{AV}_{j}}$$

(12) $${NDA}_{ij}=\frac{max(0,({{AV}_{j}-X}_{ij}))}{{AV}_{j}}$$

(13) $${PDA}_{ij}=\frac{max(0,({{AV}_{j}-X}_{ij}))}{{AV}_{j}}$$

(14) $${NDA}_{ij}=\frac{max(0,(X_{ij}-{AV}_{j}))}{{AV}_{j}}$$

*Step 4*: The weighted sum of the *PDA* and the *NDA* for each alternative are calculated as depicted in Equations (15) and (16).

(15) $${SP}_{i}=\sum_{J=1}^{m}w_{j}{PDA}_{ij}$$

(16) $${NP}_{i}=\sum_{J=1}^{m}w_{j}{NDA}_{ij}$$

*Step 5*: The weighted sum of *PDA* and *NDA* for all alternatives is normalized by using Equations (17) and (18).

(17) $${NSP}_{i}=\frac{{SP}_{i}}{{max}_{i}({SP}_{i})}$$

(18) $${NSN}_{i}=1-\frac{{SN}_{i}}{{max}_{i}({SN}_{i})}$$

*Step 6*: The appraisal scores (*AS*) of alternatives are computed by using Equation (19). Afterward, the alternatives are ranked in descending order according to the values of the appraisal score (*AS*). The alternative having the highest *AS* is considered the optimal choice among the available options.

(19) $${AS}_{i}=\frac{1}{2}({NSP}_{i}+{NSN}_{i})$$

#### 3.3.4. Technique for Order Preference by Similarity to Ideal (TOPSIS) Approach

The TOPSIS technique, a Multi-Criteria Decision-Making method, evaluates alternatives based on their proximity to the ideal best and worst values. It involves six steps, outlined as follows [61]:

*Step 1*: A decision matrix (*x_ij_*) is structured with alternatives represented in rows and criteria in columns. A significant challenge in decision-making arises from the disparate units of these criteria. To facilitate comparison among alternatives, it is imperative that the criteria either share similar units or undergo normalization. Various normalization methods exist, such as linear, non-monotonic, and vector normalization [62]. Since the TOPSIS method traditionally utilizes Euclidean distance, it employs vector normalization for criterion normalization [63]. Thus, Equation (20) is utilized to normalize this decision matrix.

(20) $$n_{ij}=\frac{x_{ij}}{\sqrt{\sum_{j=1}^{m}{\left(x_{ij}\right)}^{2}}}$$

*Step 2*: The development of the weighted normalized decision matrix (*v_ij_*) involves multiplying the weight value (*w_j_*) of the respective criterion with the matrix value (*n_ij_*), as outlined in Equation (21).

(21) $$v_{ij}=w_{j}\times n_{ij}$$

*Step 3*: For each criterion, the ideal best ($v_{j}^{+}$) and worst ($v_{j}^{-}$) values are established. When the objective is to maximize the criterion, the ideal best corresponds to the highest value among the alternatives, while the ideal worst corresponds to the lowest one.

*Step 4*: Equations (22) and (23) are used to calculate the Euclidean distance ($d_{i}^{+},\;{\;d}_{i}^{-}$) from the ideal best and worst values.

(22) $$d_{i}^{+}=\sqrt{\sum_{j=1}^{n}{\left(v_{ij}-v_{j}^{+}\right)}^{2}}$$

(23) $$d_{i}^{-}=\sqrt{\sum_{j=1}^{n}{\left(v_{ij}-v_{j}^{-}\right)}^{2}}$$

*Step 5*: Performance scores (*PS*) are computed for each alternative, indicating their relative proximity to the ideal best and worst values, through the utilization of Equation (24).

(24) $${PS}_{i}=\frac{d_{i}^{-}}{d_{i}^{-}+d_{i}^{+}}$$

*Step 6*: Criteria are ranked based on their respective *PS* values, ordered from the largest to the smallest. The alternative with the highest *PS* value is deemed as the best-performing one.

## 4. Results and Discussion

### 4.1. Criterion Weights

In the process of selecting suitable polymer materials for hybrid vehicle battery packs, the usage of the SWARA approach has facilitated a structured and quantitative approach to determining the importance of various criteria. The weights derived from this meticulous calculation method provide a deep understanding of the ranking of material properties critical for meeting the strict demands of the automotive industry. The criteria are ranked based on their importance and then their relative importance levels are determined. Then, by applying Equations (1)–(3) correctly, the criterion weights were calculated as in Table 2.

High-temperature resistance is our top priority, crucial for hybrid vehicle battery packs, carrying a 19.3% weight. It is essential for enduring extreme thermal conditions and preventing thermal runaway. Next is mechanical strength (17.55%), vital for withstanding automotive stresses like vibrations. Chemical resistance, at 11.7%, is key for enduring corrosive electrolytes, enhancing battery longevity. Wear resistance, 11.14%, is necessary for enduring operational wear, maintaining performance. The elastic modulus (9.28%) ensures materials resist deformation, vital for component alignment. Thermal expansion, weighted at 8.07%, is important for materials to minimize stress from temperature changes. Thermal conductivity (6.73%) must balance heat dissipation and insulation, optimizing battery performance. UV resistance, at 5.85%, protects against sunlight-induced degradation. Moisture absorption (5.32%) is critical for maintaining material integrity in humidity. Lastly, density (5.06%) is considered for its impact on vehicle weight but is less critical than other properties. This SWARA-based weighting guides material selection for battery packs, aligning with safety, efficiency, and sustainability goals.

### 4.2. Comparison of SWARA-Based MCDM Approaches

Upon correctly determining the criterion weights using the SWARA method, three distinct MCDM methods—namely ARAS, EDAS, and TOPSIS—were sequentially applied to ensure the selection of the suitable polymer material for hybrid vehicle battery packs. First, an initial decision matrix (see Table 3) was developed. During the development of this matrix, the value of each alternative for the criteria (i.e., maximum temperature resistance, elastic modulus, and moisture absorption rate) was considered, while alternatives for all other criteria were rated on a 5-point Likert scale (1 means lowest value and 5 represents highest value). Subsequently, the respective methodology was implemented as outlined in Section 3.3.

The MCDM methods, specifically SWARA-ARAS, SWARA-EDAS, and SWARA-TOPSIS, provide a systematic framework for evaluating and ranking thermoplastic materials for use in hybrid vehicle battery packs. The rankings (see Table 4) derived from these methods reflect a comprehensive assessment of materials based on criteria critical to the operational requirements and safety standards of hybrid vehicles. This analysis is grounded in the previously established criteria weights, emphasizing maximum temperature resistance, mechanical strength, and chemical resistance among other factors. PEEK emerges as a leading material in the rankings, consistently securing the first position in SWARA-ARAS and SWARA-EDAS and a close second in SWARA-TOPSIS. This prominence can be attributed to PEEK’s exceptional thermal stability and mechanical strength, which align with the highest weighted criteria. The material’s ability to maintain structural integrity and performance under extreme thermal conditions addresses the crucial challenge of preventing thermal runaway in hybrid vehicle battery packs. Furthermore, PEEK’s chemical resistance ensures durability against the corrosive substances within the battery environment, thereby enhancing the longevity and safety of the battery system.

**Figure 3:**
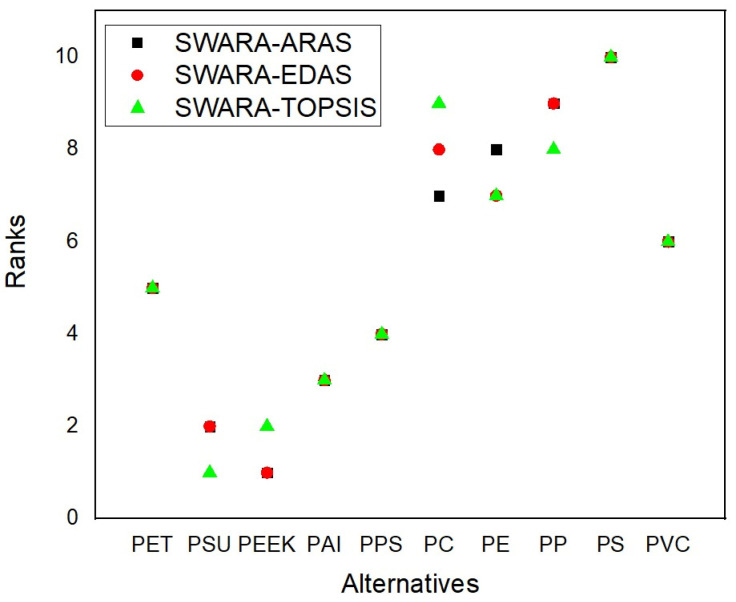
Comparison of rankings from SWARA-based MCDM methods.

PSU also demonstrates strong performance (see Figure 3) across all MCDM methods, ranking second in SWARA-ARAS and SWARA-EDAS and achieving the top rank in SWARA-TOPSIS. PSU’s high ranking is likely due to its excellent high-temperature resistance and mechanical properties, making it an ideal choice for parts exposed to elevated temperatures. This aligns with the need for materials that contribute to efficient thermal management and structural durability, ensuring the battery pack’s operational reliability under thermal stress. PAI, with consistent third-place rankings across all methods, highlights its balanced performance in key criteria areas. PAI’s high-temperature performance and dimensional stability under thermal stress make it a reliable material choice for critical battery pack components. This consistency in ranking underscores the material’s suitability for ensuring the safety and operational efficiency of hybrid vehicle battery packs, reflecting its alignment with the prioritized criteria of thermal and mechanical stability.

Polyethylene Terephthalate (PET) and Polyphenylene Sulfide (PPS) are also favorable, with PET showing good mechanical and thermal stability and PPS noted for its chemical resistance and thermal stability, which are essential for the longevity and efficiency of battery packs. These materials’ rankings across the MCDM methods validate their potential in hybrid vehicle applications, as they meet the necessary criteria of thermal management, safety, and durability. Conversely, materials like Polystyrene (PS) and Polypropylene (PP) rank lower in all methods, indicating that they may not be as suitable for the high-temperature and mechanically demanding environment of hybrid vehicle battery packs. PS, for example, has lower thermal stability and mechanical strength, making it less ideal for critical components exposed to high temperatures.

The selection process, detailed in the methodology, employs criteria such as maximum temperature resistance, mechanical strength, and chemical resistance, which are critical for ensuring the safety and performance of hybrid vehicle battery packs. The consistent rankings of materials like PEEK and PSU across different MCDM methods underscore their robustness and suitability for this application, highlighting the importance of a comprehensive evaluation in making informed material selection decisions. The detailed examination of these rankings, based on the specific criteria set for hybrid vehicle battery pack applications, shows a sophisticated method for choosing materials. By leveraging MCDM methods, this research offers a methodical framework for identifying thermoplastic materials that not only meet the technical requirements of hybrid vehicles but also correspond to broader objectives of safety, efficiency, and sustainability. This comprehensive evaluation supports informed decision-making in the material selection process, contributing valuable insights to the advancement of hybrid vehicle technology within the automotive industry.

Spearman’s rank correlation coefficient (SRCC) analysis was conducted to assess the similarity in the results generated by the various MCDM methods [64,65]. In examining the consistency of rankings generated by the three distinct SWARA-based MCDM approaches in this research, SRCCs were computed and are presented in Table 5. The correlation coefficients among all approaches exceeded 0.90, indicating a high level of agreement in results across the three MCDM methods.

Our previous study, Ordu and Der [33], focused on the selection of polymer materials for flexible pulsating heat pipe manufacturing. This current research introduces several significant scientific and thematic differences compared to the earlier work. While Ordu and Der [33] contributed to the field of materials science by proposing a comprehensive Multi-Criteria Decision-Making (MCDM) framework specifically designed for selecting polymer materials in the design of flexible fluidic systems and pulsating heat pipes, this study addresses a pressing need in the automotive industry. In particular, it focuses on the selection of polymer materials for hybrid vehicle battery packs, with an emphasis on criteria such as thermal stability and environmental impact. By employing advanced MCDM methods tailored for the automotive context, this research not only enhances the performance and sustainability of hybrid vehicles but also establishes a foundation for future investigations into emerging materials and their applications within the automotive sector. When examining the methodological differences between these two studies, several key distinctions become evident. The methodologies reflect distinct approaches to MCDM, each tailored to its respective context. Ordu and Der [33] utilized three AHP-based hybrid methods—AHP-GRA, AHP-CoCoSo, and AHP-VIKOR—which provided a structured approach to ranking polymeric materials based on fourteen evaluation criteria. This methodology highlighted the importance of comparative analysis in material selection for fluidic systems. In contrast, the present study adopts a different set of MCDM techniques, specifically SWARA-ARAS, SWARA-EDAS, and SWARA-TOPSIS, which are designed to address the unique challenges posed by high-temperature environments in hybrid vehicle battery applications. This methodology not only evaluates materials based on criteria relevant to automotive performance but also prioritizes safety and sustainability. The use of different MCDM frameworks in each study effectively addresses the specific needs and complexities of their respective fields, demonstrating the versatility of decision-making tools in material selection processes. Moreover, in Ordu and Der [33], Polytetrafluoroethylene (PTFE) was identified as the ideal thermoplastic material for flexible pulsating heat pipe manufacturing. In this study, however, Polyetheretherketone (PEEK) was selected as the optimal polymer material for hybrid vehicle battery packs. These findings highlight that different materials are more suitable for specific applications, emphasizing the importance of considering application-specific requirements in material selection. In conclusion, both studies illustrate how materials science can be applied across a wide range of engineering applications, providing tailored solutions to meet the unique demands of each field.

## 5. Conclusions

In this comprehensive study, we have explored the critical task of selecting polymer materials for hybrid vehicle battery packs in the automotive industry through a comparative MCDM approach. By systematically evaluating polymer materials against a set of rigorously defined criteria, including thermal stability, mechanical strength, chemical resistance, and environmental impact, this research has identified optimal materials that meet the demanding requirements of hybrid vehicle operations. The usage of MCDM techniques, specifically SWARA-ARAS, SWARA-EDAS, and SWARA-TOPSIS, has provided a structured framework for assessing the suitability of various polymer materials, leading to informed recommendations that prioritize safety, performance, and sustainability.

The findings of this study hold significant implications for the automotive industry, particularly in the design and manufacturing of hybrid vehicle battery packs. By identifying polymer materials that offer superior performance in high-temperature environments, this research contributes to the enhancement of battery efficiency, longevity, and overall vehicle performance. The selection of appropriate materials not only addresses the immediate operational challenges posed by hybrid vehicle battery packs but also aligns with broader objectives of reducing environmental impact and advancing sustainable automotive technologies. This work, therefore, provides valuable insights for manufacturers, guiding them in making informed decisions about material selection to improve the safety, efficiency, and sustainability of hybrid vehicles.

While this study offers a comprehensive analysis and valuable recommendations, it also acknowledges certain limitations, such as the focus on specific polymer materials and the application of particular MCDM methods. Future research could expand on this work by exploring a wider range of materials, including emerging sustainable alternatives, and by applying other decision-making frameworks to validate or complement the findings presented here. Additionally, the evolving nature of hybrid vehicle technology and the automotive industry’s shifting priorities towards more environmentally friendly solutions present opportunities for further investigation into the selection of materials that meet these new challenges. By building on the foundation laid by this research, future studies can continue to advance the field of material science in automotive applications, contributing to the development of more efficient, safe, and sustainable hybrid vehicles.

## Figures and Tables

**Table 1:** Criteria and abbreviations.

Criteria	Unit	Abbreviations
Maximum Temperature Resistance	°C	MTR
Mechanical Strength	MPa	MS
Chemical Resistance	-	CR
Wear Resistance	mm^3^	WR
Elastic Modulus	GPa	EM
Thermal Expansion	µm/m/°C	TE
Thermal Conductivity	W/mK	TC
UV Resistance	-	UVR
Moisture Absorption Rate	%	MAR
Density	kg/m^3^	D

**Table 2:** Criteria weights (%).

Criteria	*s_j_*	*k_j_*	*q_j_*	*w_j_*
Maximum Temperature Resistance		1.00	1.000	0.1930
Mechanical Strength	0.10	1.10	0.909	0.1755
Chemical Resistance	0.50	1.50	0.606	0.1170
Wear Resistance	0.05	1.05	0.577	0.1114
Elastic Modulus	0.20	1.20	0.481	0.0928
Thermal Expansion	0.15	1.15	0.418	0.0807
Thermal Conductivity	0.20	1.20	0.349	0.0673
UV Resistance	0.15	1.15	0.303	0.0585
Moisture Absorption Rate	0.10	1.10	0.276	0.0532
Density	0.05	1.05	0.262	0.0506

**Table 3:** The initial decision matrix.

		Criteria									
		MTE	MS	CS	WR	EM	TE	TC	UVR	MAR	D
Alternatives	PET	1.38	4	4	3	70	4	3	2	0.24	4
	PSU	1.24	5	5	4	55	5	4	3	0.32	4
	PEEK	1.32	5	5	5	47	5	5	2	0.25	5
	PAI	1.43	5	5	5	30	5	5	2	0.25	4
	PPS	1.35	4	4	4	55	4	4	1	0.24	4
	PC	1.20	3	4	3	65	3	3	3	0.20	3
	PE	0.96	2	3	2	160	2	2	4	0.42	2
	PP	0.91	3	3	2	125	3	2	4	0.16	3
	PS	1.05	2	3	3	75	2	2	4	0.04	2
	PVC	1.39	3	4	3	80	4	3	3	0.21	4

**Table 4:** Parameter values of the hybrid MCDM methods.

Materials	SWARA-ARAS			SWARA-EDAS				SWARA-TOPSIS			
	*S_i_*	*K_i_*	Rank	NP	NN	A	Rank	$d_{i}^{+}$	$d_{i}^{-}$	*PS_i_*	Rank
PET	0.087	0.678	5	0.143	0.847	0.495	5	0.049	0.049	0.500	5
PSU	0.106	0.831	2	0.816	0.928	0.872	2	0.036	0.075	0.672	1
PEEK	0.109	0.850	1	1.000	0.845	0.922	1	0.039	0.079	0.668	2
PAI	0.106	0.824	3	0.943	0.775	0.859	3	0.044	0.078	0.637	3
PPS	0.089	0.697	4	0.280	0.808	0.544	4	0.048	0.053	0.524	4
PC	0.079	0.613	7	0.016	0.674	0.345	8	0.060	0.035	0.364	9
PE	0.077	0.599	8	0.604	0.096	0.350	7	0.076	0.054	0.416	7
PP	0.077	0.598	9	0.300	0.350	0.325	9	0.067	0.041	0.380	8
PS	0.062	0.482	10	0.102	0.000	0.051	10	0.085	0.024	0.219	10
PVC	0.084	0.653	6	0.088	0.792	0.440	6	0.055	0.041	0.426	6

**Table 5:** Correlation analysis of SWARA-based MCDM methods.

	SWARA-ARAS	SWARA-EDAS	SWARA-TOPSIS
SWARA-ARAS	1.0000	0.9879	0.9515
SWARA-EDAS		1.0000	0.9758
SWARA-TOPSIS			1.0000

## Data Availability

Data are contained within the article.
